# Anxiety and Body Image Distress in a Type 1 Diabetes Patient With Insulin-Induced Lipodystrophy

**DOI:** 10.7759/cureus.75225

**Published:** 2024-12-06

**Authors:** Maham Tahir, Andleeb Raja

**Affiliations:** 1 Emergency Department, Bahria International Hospital, Rawalpindi, PAK

**Keywords:** acquired abdominal lipodystrophy, acquired lipodystrophy, depression and diabetes, in patient diabetes education (ide), insulin lipodystrophy, lipodystrophy negative body image, psychological aspect of diabetes, type 1 diabetes mellitus (t1d)

## Abstract

This case report presents a rare instance of a 28-year-old female patient with insulin-induced abdominal lipodystrophy, who presented to the emergency department with symptoms of an anxiety attack triggered by body image distress. She was diagnosed with type 1 diabetes at the age of eight years. For the past 10 years, she has been using insulin glargine and insulin lispro, injecting roughly five times per day. The patient knew the importance of the need to rotate injection sites and injection techniques. She mentioned that she changed her insulin needles every two weeks because she was lazy about it. She also mentioned that she used 4 mm 32G pen needles. Her diabetes was well-controlled, with glycated hemoglobin (HbA1c) levels of 6.5%. Clinical examination revealed a soft swelling with striae on the abdomen (hypogastrium and left iliac fossa). The patient reported repeated insulin injections, both rapid-acting and long-acting, in the same lipodystrophic region because it was less painful and provided easier access to the injection site in public. The patient was known to the emergency department staff due to her repeated visits with symptoms of anxiety. Lipodystrophy is a condition characterized by abnormal fat distribution at insulin injection sites. Insulin-induced lipodystrophy is a known complication of long-term insulin therapy, often leading to local adiposity, metabolic disturbances, and psychological challenges. The report underscores the importance of considering psychological aspects when managing patients with acquired lipodystrophy, highlighting the relationship between fat redistribution and depression in this patient population, who are already at increased risk of mood disorders.

## Introduction

Lipodystrophy refers to a heterogeneous group of disorders that are characterized by abnormal or absent fat distribution in the body. These disorders can either be congenital or acquired, with the acquired form most commonly seen in patients with long-term insulin use, a condition known as insulin-induced lipodystrophy. This acquired form typically manifests as fat atrophy at the insulin injection sites, often accompanied by local fat hypertrophy, insulin resistance, and metabolic disturbances such as hypertriglyceridemia and dyslipidemia, which complicate the management of diabetes [1].

While the primary concern in insulin-induced lipodystrophy is the impact on metabolic control, recent studies have also highlighted the psychological implications of the condition. Patients with lipodystrophy, particularly those who develop visible fat distribution changes, often experience significant body image disturbances, social anxiety, and depression [2].

Despite the recognition of the metabolic aspects of lipodystrophy, the psychiatric consequences are less frequently discussed, with depression often being overlooked as a consequence of the condition. Patients with lipodystrophy report having impairments in quality of life in domains such as physical health, mental health, social isolation, and stigma compared to the general population [3].

This case report aims to highlight the psychological implications of insulin-induced lipodystrophy. Through this report, we aim to raise awareness about the need for comprehensive management of insulin-induced lipodystrophy, focusing not only on glycemic control and education about injection site rotation but also on the mental health challenges faced by these individuals.

## Case presentation

**Figure 1:**
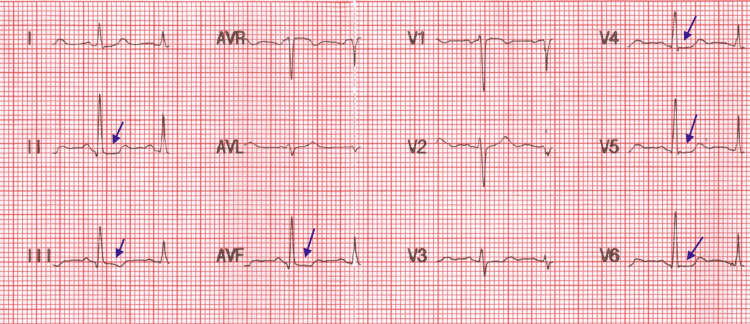
The patient's ECG on presentation showing ST segment depressions ECG: electrocardiogram

A 28-year-old female presented to the emergency department with a one-hour history of palpitations and shortness of breath. Vital signs were obtained, showing a pulse rate of 130 beats per minute (bpm), blood pressure of 140/90 mmHg, oxygen saturation (SpO_2_) of 98% on room air, random blood glucose of 160 mg/dl, and axillary body temperature of 36.5°C. She was diagnosed with type 1 diabetes 20 years ago and had been on insulin lispro and insulin glargine analog pens for the past 10 years. An electrocardiogram (ECG) was initially performed, which showed sinus tachycardia with nonspecific ST segment depressions (Figure 1). High-sensitivity troponin I and D-dimers, along with other baseline investigations, were ordered (Table 1). Although the patient did not complain of any chest pain and clearly mentioned previous episodes of anxiety attacks, cardiac and pulmonary pathology were important to rule out, given her comorbidities. The patient was also taking atorvastatin 20 mg per day for the primary prevention of cardiovascular disease (CVD), which had been started four years ago.

**Table 1:** The patient's laboratory test results HbA1c: hemoglobin A1c; Hb: hemoglobin; Trop I: troponin I; TSH: thyroid-stimulating hormone; LDL: low-density lipoprotein; HDL: high-density lipoprotein; TAG: triglycerides

Test	Patient value	Normal range
HbA1c	6.5 %	Normal: A1C below 5.7%; Prediabetes: A1C between 5.7% and 6.4%; Diabetes: A1C of 6.5% or higher
Hemoglobin	12 g/dl	12 - 15 g/dl
D-Dimer	196 ng/ml	<200 ng/ml
Trop I	0.1 pg/mL	<15 pg/ml
TSH	2 mU/L	0.45 - 4.12 mU/L
LDL	100 mg/dl	< 130 mg/dl
HDL	78 mg/dl	35 to 80 mg/dL
TAG	130 mg/dl	< 150 mg/dl
Urine albumin to creatinine ratio	20 mg/g	< 30 mg/g

Further history revealed that these anxiety attacks occurred at least once a week for the past 10 months. These attacks were associated with symptoms such as palpitations, shortness of breath, and tachycardia, which were exacerbated by her negative body image due to the visible changes caused by lipodystrophy. The patient reported significant distress related to her lipodystrophy. She felt self-conscious and disconnected from her body, which in turn triggered emotional episodes characterized by heightened anxiety.

The patient’s anxiety attacks appeared to be closely linked to her body image distress, particularly regarding her increased frustration with her changing appearance. She expressed deep frustration with the visible changes caused by lipodystrophy, which led her to avoid mirrors and rely on tight body shapers to conceal her abdominal area. These feelings of inadequacy and fear of judgment by others, including her partner, contributed to a cycle of low self-esteem, withdrawal, and anxiety. The emotional toll of these feelings, especially her fear of being judged for her physical appearance, seemed to be a significant trigger for her anxiety attacks.

The patient's history was negative for the use of stimulants (such as caffeine, cocaine, or certain medications) or withdrawal from substances (including alcohol or benzodiazepines), ruling out these factors as potential contributors to her anxiety attacks.

While the patient's anxiety could be considered generalized, the frequent occurrence of symptoms triggered by body image concerns suggests that the anxiety may be primarily driven by distress over physical appearance. The anxiety attacks appeared to be manifestations of body image distress, likely exacerbated by the physical changes induced by lipodystrophy. The Diagnostic and Statistical Manual of Mental Disorders, Fifth Edition (DSM-5) criteria for anxiety disorder were fulfilled based on the patient's persistent symptoms of excessive worry, physical manifestations of anxiety (such as palpitations, tachycardia, and shortness of breath), and disruption of daily functioning [4]. These symptoms aligned with anxiety disorders in general, though the patient's distress is notably influenced by the changes in her appearance due to lipodystrophy.

**Figure 2:**
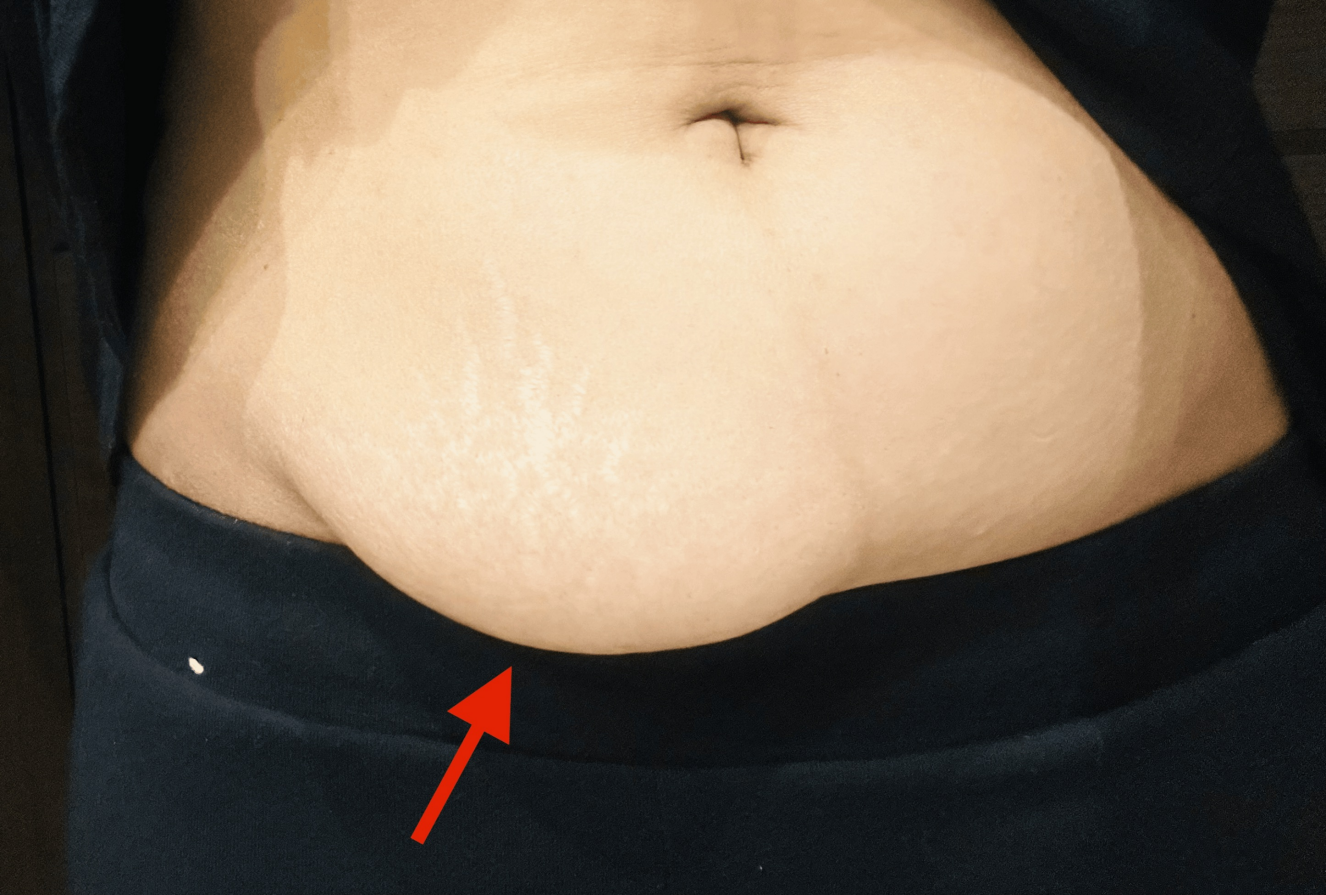
Insulin-induced abdominal lipodystrophy noted during clinical examination

Clinical examination revealed a soft swelling with striae on the abdomen (hypogastrium and left iliac fossa) (Figure 2), self-inflicted cut marks, and a pulse consistent with regular tachycardia. Chest auscultation revealed bilateral equal air entry.

**Figure 3:**
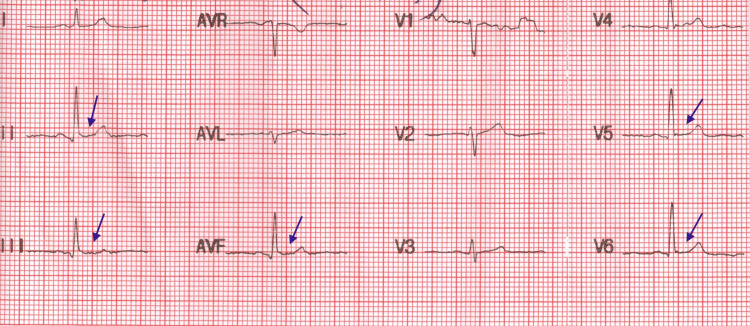
ECG changes showing improvement 40 minutes after the administration of tablet alprazolam (0.25 mg) and tablet propranolol (10 mg) ECG: electrocardiogram

An ECG was repeated 40 minutes after administering one tablet of alprazolam 0.25 mg orally and one tablet of propranolol 10 mg orally. The ECG changes had improved (Figure 3). The next day, a 2D echocardiogram and exercise tolerance test were also performed in the outpatient department (OPD).

The Generalized Anxiety Disorder Scale-7 (GAD-7) was scored at 16, classifying it as severe anxiety. The Patient Health Questionnaire-9 (PHQ-9) was scored at 18, classifying it as moderately severe depression.

Two-dimensional echocardiography revealed normal left ventricular size and systolic function. No significant valvular abnormality was detected. The exercise tolerance test was negative for ischemia.

This 28-year-old woman expressed deep frustration with the visible changes caused by lipodystrophy (Figure 2), particularly the difficulty in wearing certain clothes. She relied on tight body shapers whenever she went out, feeling the need to conceal herself. She avoided looking in the mirror without clothes, feeling disconnected and unhappy with her reflection. The patient mentioned that she was satisfied with her physical appearance until the lipodystrophy worsened. Her body image struggles also impacted her relationship. She had withdrawn from physical intimacy due to a fear of being judged by her partner. The emotional toll of these feelings contributed to a cycle of low self-esteem and withdrawal from activities she once enjoyed, causing an impact on her daily life, including social, occupational, and personal functioning.

Body dysmorphic disorder (BDD) was ruled out in this case. The patient did not demonstrate delusional preoccupation or obsessive focus on perceived flaws to the extent seen in BDD. While she expressed frustration and distress over her appearance, these feelings were more situational and linked to the visible effects of lipodystrophy rather than a disordered perception of body image typical of BDD. Social anxiety disorder, post-traumatic stress disorder (PTSD), and obsessive-compulsive disorder (OCD) were ruled out based on the patient's history and lack of specific symptoms associated with these conditions.

Management included sessions with a diabetes nurse for proper guidance on injection rotation, choosing the right-sized needle, and injection techniques. Cognitive behavioral therapy was also advised. The patient was commenced on sertraline 50 mg per day. Insulin glargine and insulin lispro were continued as a basal-bolus regimen. Additionally, 20 mg of atorvastatin per day was continued as before.

## Discussion

This case highlights the important relation between insulin-induced lipodystrophy and its psychological impact. Lipodystrophy is a condition marked by abnormal fat distribution at insulin injection sites; it is not only a physical complication of insulin therapy, but it can also lead to significant psychological distress. The patient in this case reported using the same insulin needle for up to two weeks and failing to rotate injection sites due to convenience and discomfort, which likely contributed to the development of lipodystrophy (Figure 2). Frequent use of the same needle and lack of injection site rotation are known risk factors for the development of insulin-induced lipodystrophy [5]. Patients often report negative body image, and those living with lipodystrophy experience reduced quality of life compared to the general population. The QuaLip study demonstrates that lipodystrophy is associated with poor health-related quality of life scores, worsened by an increased prevalence of psycho-emotional disturbances such as anxiety and depression [6]. The patient in this case, a 28-year-old woman with type 1 diabetes, struggled with anxiety and low self-esteem related to her body image. This is consistent with findings from other studies that suggest individuals with lipodystrophy frequently experience mood disorders, anxiety, and eating-related disturbances [7].

The psychological distress seen in patients with lipodystrophy highlights the need for an integrated care approach that addresses both the physical and mental health aspects of the condition. In this case, the patient was treated with sertraline, a selective serotonin reuptake inhibitor (SSRI), which has been shown to be effective in managing anxiety disorders. A study demonstrated that sertraline is both effective and well-tolerated in treating anxiety, which aligns with the management approach in this case [8]. Furthermore, psychological support, such as cognitive behavioral therapy, is recommended to address body image issues [9]. This case highlights the importance of considering the psychological needs of patients alongside the physical treatment of diabetes-related complications.

In conclusion, the psychological impact of insulin-induced lipodystrophy is often under-recognized. Studies indicate that patients with lipodystrophy face significant emotional challenges, including anxiety, depression, and a distorted body image [10]. Healthcare providers should consider these psychological aspects when managing lipodystrophy, ensuring that comprehensive care includes both metabolic management and psychological support to improve overall well-being.

## Conclusions

This case highlights the importance of addressing the psychological aspects of insulin-induced lipodystrophy. While the physical manifestations of lipodystrophy, such as abnormal fat distribution and injection site complications, are well-documented, the associated psychological burden, including anxiety, body image issues, and feelings of distress, are significant and warrant attention. In this case, the patient’s anxiety attacks were compounded by concerns over her appearance and fear of judgment, which negatively impacted her daily functioning and well-being. It is crucial to emphasize education on proper injection techniques, including rotation of injection sites, to prevent further complications. Furthermore, addressing the psychological aspects of this condition through psychosocial support, such as cognitive-behavioral therapy and, if appropriate, pharmacotherapy, can be beneficial in improving the patient's mental health. A multidisciplinary approach is essential to ensure that both the physical and psychological needs of patients with insulin-induced lipodystrophy are addressed.
